# SIFT Based Vein Recognition Models: Analysis and Improvement

**DOI:** 10.1155/2017/2373818

**Published:** 2017-06-07

**Authors:** Guoqing Wang, Jun Wang

**Affiliations:** School of Information and Electrical Engineering, China University of Mining and Technology, Xuzhou, Jiangsu 221006, China

## Abstract

Scale-Invariant Feature Transform (SIFT) is being investigated more and more to realize a less-constrained hand vein recognition system. Contrast enhancement (CE), compensating for deficient dynamic range aspects, is a must for SIFT based framework to improve the performance. However, evidence of negative influence on SIFT matching brought by CE is analysed by our experiments. We bring evidence that the number of extracted keypoints resulting by gradient based detectors increases greatly with different CE methods, while on the other hand the matching result of extracted invariant descriptors is negatively influenced in terms of Precision-Recall (PR) and Equal Error Rate (EER). Rigorous experiments with state-of-the-art and other CE adopted in published SIFT based hand vein recognition system demonstrate the influence. What is more, an improved SIFT model by importing the kernel of RootSIFT and Mirror Match Strategy into a unified framework is proposed to make use of the positive keypoints change and make up for the negative influence brought by CE.

## 1. Introduction

**Figure 1:**
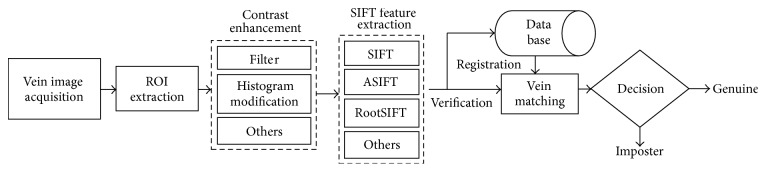
General framework for local invariant feature based vein recognition system.

Vein recognition has emerged as a new biometric trait for accurate and fast people identification recently and has received growing attention as a result of live-body and anti-interference identification, simple-acceptability, and anticounterfeit pattern [1]. A general framework for vein recognition usually refers to preprocessing (CE), feature extraction, and matching. Among numerous researches for reliable vein recognition, nearly half of them focus on robust feature extraction model design from different perspective, for example, the famous curvature information [2–4] and Gabor filter design [5–7] to obtain the distinguished geometry-based feature; the classical LBP [8] and image invariant moment [9, 10] method to represent the statistical feature; the SIFT or SURF [1, 6, 11–21] for local invariant feature extraction. To construct a less-constrained vein recognition system which renders no restriction on hand gesture and location, distance of hand from capturing device, only the local invariant feature based system is effective. However, if the local invariant features are directly extracted from the images directly, it is difficult to obtain sufficient keypoints because vein imaging under near-infrared (NIR) illumination usually appears dark and of low contrast [1]. To address this problem, a necessary preprocessing step referring to contrast enhancement is included in all the related SIFT/SURF feature based vein recognition system [1, 13–21], all of which share the consistent framework as illustrated in Figure 1.

It will be induced to perceive that contrast enhancement is realized by sharpening the useful information while compressing the background information, which will result in the grey value gradient change and bring about great impact on local invariant features detection based on the gradient analysis. However, it is well known that a great majority of the enhancement methods is not robust to illumination change existing in vein image acquisition, which indicates that CE will result in different output for the same person with images captured at different period. The performance of SIFT will be influenced because the robustness of keypoints detection and description depends greatly on the properties of the object image. However, to date only few researchers publish results that address the impact of contrast enhancement over the SIFT extraction and description procedure. For example, Dharavath et al. [22] come to the conclusion that a cascade of 2-D preprocessing would enhance the performance of SURF based image matching task. Stanciu et al. [23] brought evidence that the number of keypoints that can be automatically extracted by gradient based detectors increased with CE, and that matching gradient based keypoints descriptors extracted from processed image sets by CE is negatively affected in terms of precession-recall. Kalia et al. [24] have shown that CE brings great improvement in feature detection repeatability by conducting experiments with various state-of-the-art detectors, while Campos et al. [25] concluded that the adopted Gabor filter did not bring any improvement in the case of SIFT based ocular recognition system. The experimental result of vein recognition system that we present sheds more light in this poorly explored field, figuring out that CE for obtaining visually clearer vein distribution comes accompanied with side effects in respect to the detection and matching of SIFT. To be specific, the number of keypoints is increased greatly while the matching procedure of SIFT is negatively affected in terms of Precision-Recall and EER when both the registered and verified samples are previously processed by CEs. It is of great significance for our findings because the core of SIFT based vein recognition system is based on determining interest point correspondences between individual image pairs (Verification) or between an image and a class of images (Identification), in which the fluctuations in the number of the extracted keypoints or in the robustness of the SIFT descriptor generation and matching would bring the performance down.

To sufficiently demonstrate the specific influence of CEs, nearly all CEs adopted in the aforementioned SIFT based vein recognition system are reexperimented, followed by SIFT feature extraction and matching to evaluate the specific influence of CE on the keypoints detection and matching, which reflects in number change and PR/EER, respectively. The referred CEs are, respectively, HE (histogram equalization) [12, 18], IN (intensity normalization) [13, 16], IHE (illumination estimation subtract and HE) [14], DHE (DoG filter and HE) [15], HF (homomorphic filter) [16], GC (gamma correction) [16], RASF (Retinex and adaptive smoothing filter) [17], CLAHE (contrast limited adaptive histogram equalization) [19], related AHE (adaptive histogram equalization), CLHE (contrast limited histogram equalization), HHE (high frequency filtering and HE) [20], INE (image negative enhancement) [21], GLS (grey level slicing) [21], CS (contrast stretching) [21], LS (Laplacian sharpening) [21], UM (unsharp masking) [21], HBF (high-boost filtering) [21], and HEHBF (HE and HBF) [21]. To the best of our knowledge, all the listed CE turned out enhancing the final SIFT based vein recognition system performance in terms of EER. Carried with the confidence that performance will be improved [12–21] and with the reality that performance will be kept unchanged or declined [22–25], it is necessary to conduct comprehensive experiment to find out the specific influence of CE on SIFT based vein recognition system.

The paper is divided into two main parts: the first of which refers to experiment and result concerning the specific impact introduced in Sections 2 and 3; the second part of the paper covers how to overcome the influence based on the proposed model that combines the kernel of RootSIFT and new matching strategy as MM, which is introduced in Section 3, while in Section 4 we outline our conclusion.

## 2. How CE Influences the SIFT Based Vein Recognition System

This section is organized as follows: in Section 2.1, we briefly introduce and discuss the key concepts of the eighteen CE algorithms which we have tested against SIFT, while in Section 2.2 the image set we adopted in the experiment design is present.

### 2.1. Evaluated Contrast Enhancement Models

To efficiently demonstrate the impact of CEs toward SIFT detection and the following matching of SIFT descriptor, eighteen relatively simple but state-of-the-art enhancement methods are briefly introduced in the following paragraphs.

To the best of our knowledge, we tend to summarize the adopted CEs in SIFT based vein recognition system into three main groups: the first one refers to those which focus only on the image itself and changing the pixel grey value by adopting linear/nonlinear function; on contrast, the second one involves the effective and simple models based on the analysis of histogram information while the third CE model improves the performance of HEs by adding transformations to the input subject of HEs so as to obtain better results with enhancement on useful detailed information.

Linear/nonlinear functional enhancement (LNFE): IN is one of the most cited preprocessing methods to deal with the case in which lighting condition changes are unavoidable [26]. A general framework for intensity normalization is to transfer all the to-be-processed images into the one with the same mean and variance, and then the images formed under overlighting or weak-lighting condition would be processed into better visual effect. The similar condition with IN: other function based CE belonging to the first group is a point function, which realizes the slicing of specific grey values from the rest of the grey values for enhancement in GLS [27]. Besides, CS tries to span the range of pixel's intensity to desired range of values, which is implemented by specifying the desired minimum and maximum value limits [28]. More professionally, the gamma correction curve [29] is built based on the classical gamma function to choose the best distribution one and then fit the distribution of the original image to the selected curve to obtain the gamma corrected image which is enhanced in detailed information. On the other hand, filter processing for image enhancement is also directly conduct transformation on the original image. For instance, the LS [21], which is a second-order derivative method, is realized by constructing Laplacian filter to find fine details of an image and restore the fine detail to obtain enhanced result. Similarly, the unsharp filter [30] for enhancing edges and high-boost filter [30] for enhancing the high frequency component while keeping the low frequency components are also realized to examine the specific influence.

HE and its variants (HEs): HE is one of the most commonly used methods for image CE because of its high efficiency and simplicity. The specific histogram equalization is achieved by transforming a pixel value using its corresponding cumulative distribution function such for obtaining resulted images taking over a uniform distribution of intensity [31]. However, it will be less effective when the statistical contrast characteristics vary across the overall image. Adaptive HE [32–34] is proposed to overcome this drawback through generating the mapping function for each pixel from the neighbourhood content based histogram. What is more, the AHE does not allow the degree of CE to be regulated, the extent to which the character of the processed image is changed into undesirable result. Another transformation of HE to avoid the unexpected intensity change is CLHE [35], which add restriction item in the histogram transformation framework. One of the most successful and widely applied HE transformations is CLAHE, which first separates the image into various of continuous and nonoverlapped subblocks; then every subblock is enhanced individually followed by an designed interpolation operation to reduce the block artefacts [34].

Hierarchical enhancement models (HEM): the third group, which we prefer to call MLE-HE (multilayer enhancement), usually performs better than the other basic ones in case of images with less-partition useful information. The HEHBF [30], which combines high-boost filter with HE, achieves unsatisfactory result because the HBF process on HE would filter some useful information which is adjusted to a more consistent distribution after HBF process, while the HHE [20] obtains the similar result by adopting high frequency emphasis filter to take place of the HBF. To improve the performance of the mixed enhancement model, the RASF, which combines the Retinex and filter together, is introduced in [17]. The Retinex [36] is firstly realized to remove the influence of illumination and obtain the reflective nature of the hand vein images, followed by iterative adaptive smoothing filtering process to obtain better smoothing and enhancement result. To better present the detailed information and increase the number of detected keypoints, Kang et al. [1] proposed a hierarchical enhancement model on the basis of Lowe's work [37]; the model firstly adopted DoG (difference-of-Gaussian), which is a band-pass filter that discards all but a few spatial frequencies that are present in the original grey images, to get the preliminary-enhancement result by subtracting one blurred version of an original image from another less blurred one. Then the HE is used to increase the contrast and to highlight the detailed vein structure.

As mentioned above, all the enhancement methods are designed to increase the performance of SIFT in respect to the keypoints detection increase and better PR/EER [23].

### 2.2. Experimental Image Set

**Figure 2:**
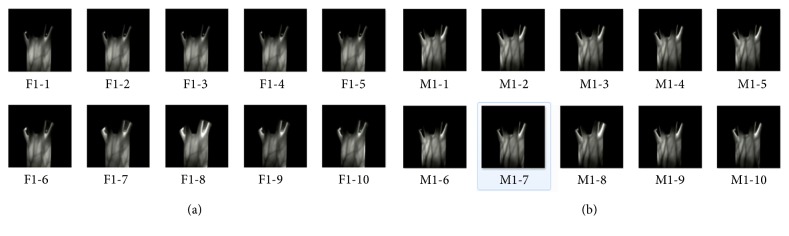
Sample of the lab-made database. (a) Female example and (b) male example.

To reliably evaluate the influence of CE on SIFT, experiments of keypoints detection and matching in terms of SIFT are conducted with trial of eighteen CEs. Before experiments, a comprehensive hand-dorsa vein database is built and experimented. In our database, 2000 sample images were acquired from the left and right hands of 100 subjects covering male (students and teachers) and female (students and teachers). Ten different images from each hand represent ten different capturing conditions diverse in illumination, capturing time (morning, noon, or afternoon). All images in the database were acquired in two specifically set sessions separated by a time interval of more than 10 days, and, at each time, five images were acquired from each hand at the wavelength of 850 nm. To the fullest of the dorsal vein information, we set the size of the images as 460*∗*680 with extremely high-quality.  Figure 2 illustrates samples of the lab-made database. Note that all the images used for CE and SIFT detection are the ROIs of the original captured ones; the specific ROI method is realized according to [38].

Several measurements were adopted to evaluate the specific influence of CE on SIFT model, including the PR [23] (Precision-Recall) and EER [1] (Equal Error Rate). The PR value represents the precision (positive predictive value) against the recall (true positive rate) and is equivalent to the false discovery rate value, whereas the EER value locates in the points where the false rejection ratio equals the false acceptance ratio in ROC (receiver operating curve). Besides, the statistical number change of detected keypoints is also the benchmark for evaluation.

### 2.3. Experimental Results and Discussion of CEs on SIFT

This section is structured as follows: in Section 2.3.1 the results concerning how the related eighteen CEs influence the detection of SIFT are presented; in Section 2.3.2 we refer to the performance change of the same eighteen CE techniques on the matching of SIFT descriptors while the further discussion about the achieved results and specific implications are presented in Section 2.3.3.

#### 2.3.1. Experimental Results and Discussion of CEs on SIFT

**Figure 3:**
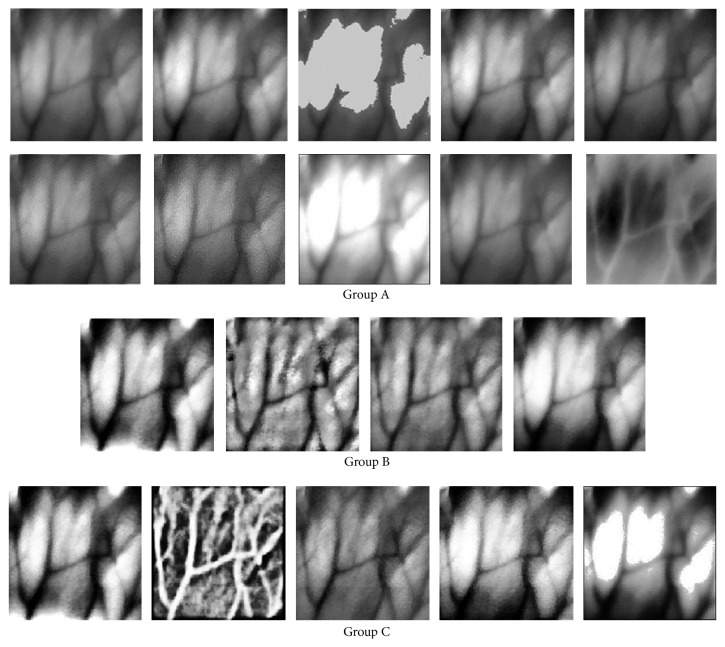
Illustration of the images after different CEs process: (A) CE processed by LNFEs including IN, GLS, CS, GC08, LS, UM, HBF, HF, and INE, respectively (the first one is the original vein image); (B) CE processed by HEs including HE, AHE, CLAHE, and CLHE, respectively; (C) CE processed by HEM including IHE, DHE, RASF, HHE, and HEHBF, respectively.

Prior to the robust SIFT keypoints detection stages the established vein image sets are preprocessed for contrast enhancement by LNFE methods: IN, GLS, CS, GC, LS, UM, HBF, HF, INE, and DoG, by HEs methods: HE, AHE, CLHE, and CLAHE, and by HEM methods: IHE, DHE, RASF, HHE, and HEHBF. Due to the limited-space consideration, only one example to illustrate the keypoints change with sampled image is shown in Figure 3, while the overall results that we present in Table 1 refer to the average values obtained for the eighteen CEs situation. There is no doubt that the specific individual results for each of the evaluated CEs scenarios follow the same trend as stated in Table 1.

**Figure 4:**
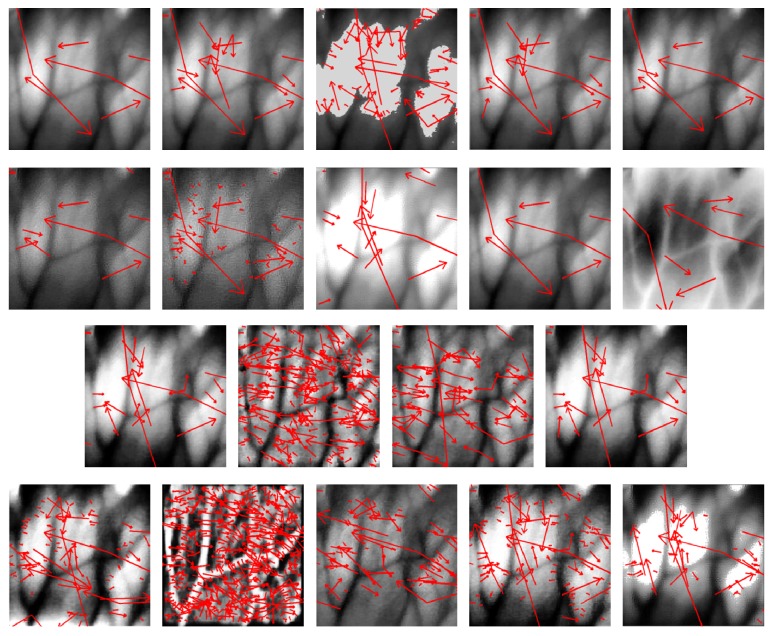
Illustration about the variations of SIFT keypoints detection with different CEs. (The listing queue of figures is as that in Figure 3.)

For each of the original image set processed after certain CEs, the SIFT keypoints were extracted by using the detectors originally reported in [37]. We observed that preprocessing an vein image for effect of CE by all listed methods belonging to LNFE, HEs, and HEM except GC, HF and INE of LNFE yield an extraordinary high number of detected SIFT keypoints than the case in which no such transformations are performed. The described situation can be observed in Table 1 and Figure 4, where we display the keypoints increase in the case of the evaluated lab-made image sets. The times by which the keypoints change is illustrated in Table 1 covers a comparison in the number of keypoints between the cases when the keypoints are extracted from the original images and when they are extracted from the images preprocessed by respective CEs. Driven by in-depth analysis on the specific content evolution before and after CEs, it can be concluded that the image pixel values are remapped to a corresponding wider range of grey level values when the CEs are performed. With the increase of pixel value range, the probability of difference between neighboring pixels is also increased, thus resulting in an increase in the possibility of identifying scale-space extrema, followed by increase in the number of detected keypoints.

The focus on the number of the table is the changing trend and increases amplification of the keypoints in respect to different CEs but not the specific change with male or female, with right hand or left hand. Consequently, the analysis on the number change is taking the CE methods as subject where we choose the best result of one CE method on the four listed subjects (FL, FR, ML, and MR). Among the LNFE methods that we tested, UM yields the highest increase in the number of detected SIFT keypoints, with average times of 7.6 for all the evaluated vein images no matter the hand or gender, followed by GLS with 5.4. Similar with LNFE methods, the HEs renders no great improvement on the number of detected keypoints with AHE achieving the highest one 28.1. Higher than majority of the former kinds of CE methods, HEM yields extraordinary increase, in which the highest was with 39.9 and the lowest one was with 5.8, respectively.

Generally speaking, the increase in the number of automatically detected SIFT keypoints would be typically regarded as a positive effect in the case of most computer vision applications. However, it is reported in [23] that matching gradient based keypoint descriptors extracted from image sets preprocessed by CE are negatively affected in terms of Precision-Recall. As a result, an assumption that the EER of SIFT based vein recognition system would also be affected in a similar way is made, which sets out a question “is contrast enhancement necessary for vein recognition system based on local invariant feature?”; the following matching experiment would figure out the answer in respect to the specific vein recognition task with the selected CEs.

To further verify the influence of different CEs on keypoints detection, the palm vein of PUT Vein Database [39] is also adopted for verification and comparison, and the specific changing trend is illustrated in Table 2.

Judging from the results as illustrated in Table 2, it could be concluded that the influence brought by contrast enhancement on SIFT keypoints detection is reliable and positive, which further demonstrates the necessity of incorporating CE into the SIFT based framework (Figure 1) for better performance. As for the specific changing results, UM realizes the highest increase in the number of detected SIFT keypoints, with average times of 7.8 for all the evaluated vein images whatever the capturing session. Regarding the HEs methods, AHE achieves the highest one with average times of 29.3 while the other three ones render no great improvement. Higher than majority of the former kinds of CE methods, HEM yields extraordinary increase, in which the highest one is with 38.7 and the lowest one is with 6.3, respectively.

#### 2.3.2. Effects of CEs on the Matching of SIFT Descriptors

**Figure 5:**
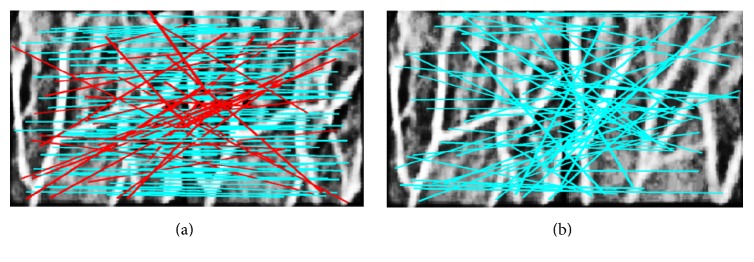
Illustration of intra- and outer-matching under the preprocessing of DHE. (a) MM of IM and (b) OM.

Before detailed description of the corresponding trend of PR/EER in respect to different CEs, some situations are defined in respect to the PR experiment and EER experiment. In the PR scenario, TM is the total matching and MM is defined as the mismatching as shown in Figure 5(a), both of which are conducted within two same-subject vein images, while on the other hand in the EER scenario, IM represents matching between vein images belonging to the same person and OM corresponds to the different subject situation as shown in Figure 5(b). It should be noted that different distratio will result in different matching result as the results shown in Figure 5 are obtained with distratio set as 0.7. Consequently, it is necessary to tell how the distratio, defined as a threshold to determine whether a matching is needed to be saved or not, is set appropriately to obtain the best matching performance for a specific CE itself.

**Figure 6:**
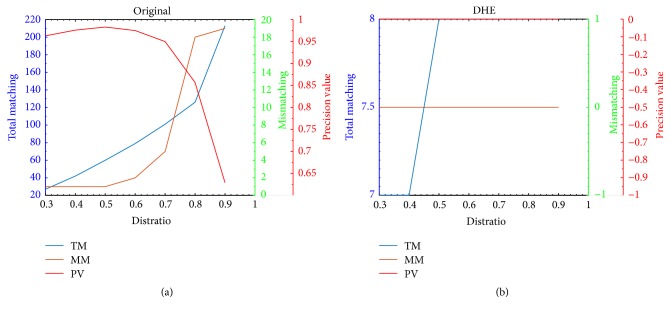
Change of TM, MM, and PV with different distratio set. (a) Before CEs and (b) CE processing by DHE.

In this paper, the discussion about distratio is independent to different evaluation item including PR/EER. The PR only focuses on the intragroup experiment, and the trend of TM/MM/PV (precision value calculated by (1)) change in respect to DHE is as shown in Figure 6(b). It can be concluded from Figure 6(b) that both the TM and MM increase with the distratio while the PV shows another trend by increase (distratio: 0.3–0.5) followed by decrease; to effectively demonstrate the influence of CEs in respect to PR, the best distratio set is determined according to the highest value of PV, and the optimal distratio for other CEs is also determined as DHE. It should be noticed that different optimal distratio is set for different CEs in this paper. Besides, it can be observed that even the optimal distratio set will result in mismatching while the matching experiment without any enhancement process does not render mismatching as shown in Figure 6(a), which indicates that the CEs will result in a lower PR.

(1) $$\begin{array}{c} PV=\frac{\#TM-\#MM}{\#TM}. \end{array}$$

**Figure 7:**
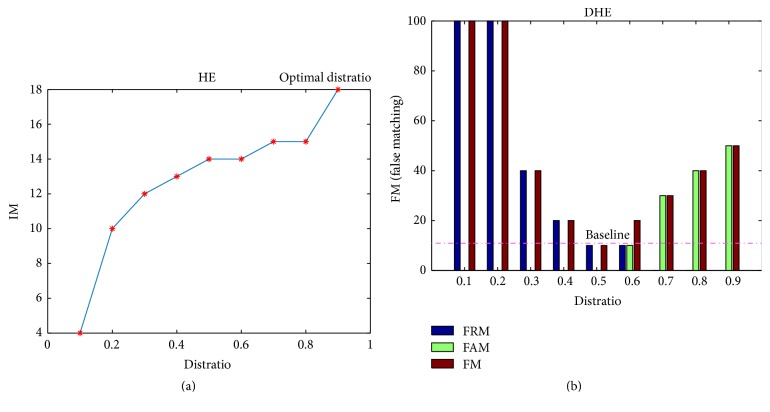
Change of matching points with different distratio. (a) Situation without FRM and FAM. (b) Situation with FRM and FAM.

In the case of EER experiment, two new matching situations are introduced including the FRM and FAM. The FRM (false rejection matching) is the situation in which the minimum matching keypoints is less than the predefined threshold (TH) determined by the optimal distratio in respect to CE, while the FAM (false acceptance matching) is the one where maximum matching keypoints appear larger than the predefined TH. The optimal distratio determination of EER could be classified into two conditions as shown in Figure 7. The first is the matching condition in which the FRM and FAM do not exist, and the optimal distratio corresponds to the one resulting in the highest IM regardless of the MM, and the optimal one corresponds to the highest point in Figure 7(a) under the preprocessing with HE. In contrast, there exist FRM and FAM in the second condition, and the related experiment is conducted in selected 100 samples with 10 subjects (random selection for left or right hand). The optimal distratio is the one resulting in lowest times of FRM and FAM in mode of 1 : 100 matching as shown in Figure 7(b), and FM represents the total number of FRM and FAM.

**Figure 8:**
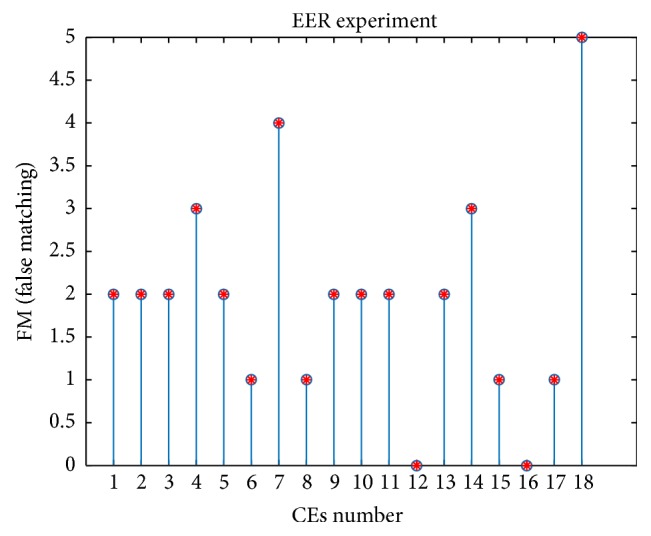
False matching value of different CEs with the optimal distratio set. CEs number 1 : 18 represents IN, GLS, CS, GC08, LS, UM, HBF, HF, INE, HE, AHE, CLAHE, IHE, DHE, RASF, HHE, and HEHBF, respectively.

It can be observed that the optimal distratio for both situations as shown in Figure 7 can be uniquely determined. By the above approach, the optimal distratio for all the eighteen CEs is determined by the similar way, followed by conducting the matching experiments to obtain the specific distribution of FRM and FAM as shown in Figure 8. Negative conclusion with PR experiment could be obtained that the CEs will undoubtedly bring down the EER as a result of generating false matching no matter if it is FRM or FAM.

After detailed analysis on how the CEs will affect the PR and EER, the overall decreasing result of PR/EER when compared with the original matching experiment is as shown in Table 3.

Observing the increase in the total number of detected SIFT keypoints that accompanies CE, it will be inclined to think that such an increase as shown in Table 1 would result in a boost-up in the matching of SIFT keypoint descriptors calculated from image pairs that have been subjected to CEs. However the PR/EER analysis on the matching of SIFT shows that in fact the increase in the extracted number of keypoints comes accompanied instead by a decrease in the performance of the matching procedure.

It should be noted firstly that the changing trends of PR and EER keep consistent with each other. Among the LNFE methods that we tested, UM yields the highest decrease in the descriptor matching of detected SIFT keypoints, with −11.76% and −2.25% for PR and EER, respectively, followed by GLS with −9.16% and −2.11%. Unlike LNFE methods, both the HEs and HEM result in more severe decrease in the matching experiment. The HEM renders generally higher increase on the SIFT descriptors matching with DHE achieving the highest one −37.29% and −7.97%. A little bit lower than the HEM, HEs also yield extraordinary decrease, in which the highest was with −23.36% and −5.14% while the lowest one was with −8.11% and −1.91%, respectively.

Similar with the experiment setup in keypoints increasing evaluation, the same recognition experiment is conducted with the PUT Vein Database [39] for verifying the conclusion, and the specific EER and PR changes could be referenced from Table 4.

Comparing the results in Table 4 with that in Table 3, it could be concluded that the changing trend in terms of PR and EER is nearly the same, despite little difference with HEM models, where the HHE renders the most significant negative influence with PUT database while the one on the lab-made database is DHE. Whatever the slight difference, the most important conclusion could be obtained from results in Tables 3 and 4 that the contrast enhancement has negative influence in keypoints matching, which is different from that with keypoints detection.

#### 2.3.3. Discussion

**Figure 9:**
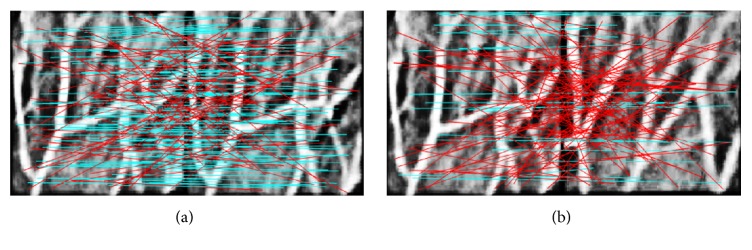
Matching results in terms of DHE. (a) Intramatching and (b) Outer-matching. The cyan lines represent the MM, where the two matching points hold different coordinates, while the red lines represent the correct matching.

The aim of the experiments of this part is to show that the adopted CEs influence the extraction of SIFT keypoints and also their description and matching. The achieved results show that contrast enhancement, which raises the dispersion of the pixels, yields a stronger answer to gradient operators which in turn results in a considerable increase in the number of detected SIFT feature. The key is that this situation can be regarded as potentially advantageous for vein recognition, for which the number of extracted features plays an important role. However, our experiments with vein recognition task show that CE is associated with a decrease in the performance of nearest-neighbour (NN) matching of keypoint descriptors, which consequently bring the PR/EER value down. The case of bringing down the PR is realized by importing the MM (mismatching) in the intragroup matching experiment as shown with the red line in Figure 9(a), while the case of EER decrease is owing to the generation of OM (outer-matching) as a result of increasing the number of keypoints by CEs as shown in Figure 9(b).

As for the reason why there exists the negative influence on PR/EER, it can be analysed as follows: in the case of NN matching in SIFT framework, the matching of true positive [23] depends on the similarity of the descriptor vectors of keypoints extracted from corresponding physical locations in two vein images as illustrated in Figure 9(a); a higher degree of similarities between the descriptors with different physical location would be resulting by CEs, which is likely to interfere with the performance of the NN matching. Moreover, CEs can increase the amount of keypoints extracted from the neighbouring physical locations, which would also interfere with the descriptor matching procedure because of the fact that the similarity of the neighbouring descriptors can be high [23]. Furthermore, CEs may increase the similarity between keypoints descriptor due to specific content transformations.

On the one hand, the experiments well demonstrate the negative influence of CEs on the performance of PR/EER of vein recognition task along with the advantage of increasing the amount of detected SIFT keypoints. On the other hand, a question arises that why every published vein recognition system [13–21] announces higher EER result with defining the CEs as the essential part. With this question, we try to find the way by which we could not only take full use of the increase in keypoints but also obtain a higher EER as reported in [13–21].

## 3. How to Overcome the Negative Effect of CE on SIFT Based Vein Recognition System

This section is organized as follows: in Section 3.1 we briefly introduce the theory of MM and Kernel Change of RootSIFT; in Section 3.2 the recognition experiment with the improved SIFT algorithm is presented while the further discussion about the achieved results and specific implications are presented in Section 3.3.

### 3.1. Introduction to Kernel Transformation and MM

#### 3.1.1. Theory of Hellinger Kernel

In the design of RootSIFT [40], a new strategy by mapping the Euclidean distance to the Hellinger kernel is proposed to increase the distinctiveness of the feature vectors.

The traditional equation for calculation of Euclidean distance is defined as

(2) $$\begin{array}{c} D\left(\;x_{i},y_{j}\;\right)=\sqrt{{\left(x_{i}-x_{j}\right)}^{2}}+\sqrt{{\left(y_{i}-y_{j}\right)}^{2}}\;x,y\in R. \end{array}$$

And the Hellinger kernel for two *L*_1_ normalized vectors is defined as

(3) $$\begin{array}{c} H\left(\;x,y\;\right)=\overset{n}{\underset{i=1}{\sum }}\sqrt{x_{i}y_{i}}\;\overset{n}{\underset{i=1}{\sum }}=1,\;\;x_{i},y_{i}\geq 0. \end{array}$$

In RootSIFT, the traditional feature descriptor is firstly normalized to a Euclidean unit vector to maintain the invariant property. Thus, the specific relationship between the Euclidean distance and Hellinger kernel is defined as follows:

(4) $$\begin{array}{c} d_{E}{\left(\;x,y\;\right)}^{2}={\left\|\;x-y\;\right\|}_{2}^{2}={\left\|\;x\;\right\|}_{2}^{2}+{\left\|\;y\;\right\|}_{2}^{2}-2x^{T}y, \end{array}$$

where the key kernel is defined as

(5) $$\begin{array}{c} S_{e}\left(\;x,y\;\right)=x^{T}y. \end{array}$$

And then the relationship function can be rewritten as follows:

(6) $$\begin{array}{c} d_{E}{\left(\;x,y\;\right)}^{2}=2-2S_{e}\left(\;x,y\;\right). \end{array}$$

The key of Hellinger kernel for similarity measurement is the implementation of two algebraic operations: (1) normalize the original 128 × *N* feature vector to *L*_1_, which is originally unitized to *L*_2_ norm; (2) figure out the square root of each element in the normalized vector. The specific transformation could be defined as follows:

(7) Sex,y=xTy=∑i=1nxiyi=Hx,y,Sex,x=xTx=∑i=1nxi=1.

Therefore, the improved Euclidean distance could be redescribed based on the transformation of Hellinger kernel:

(8) $$\begin{array}{c} d_{E}{\left(\;\sqrt{x},\sqrt{y}\;\right)}^{2}=2-2H\left(\;x,y\;\right). \end{array}$$

After obtaining the Hellinger kernel based feature vector representation, the experiment would go on to the NN matching procedure.

#### 3.1.2. Theory of Mirror Matching

**Figure 10:**
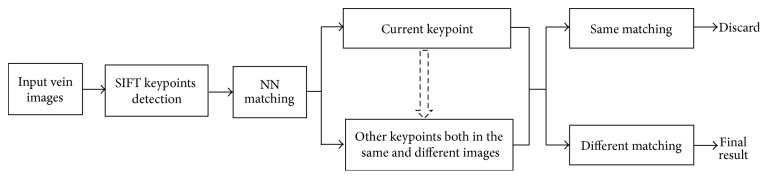
Matching diagram of MM framework.

The generation of MM [41] is inspired by a simple but novel idea: if a given feature point in one image is better matched with other points from the same vein image than points in the other image, then any matches from this feature point to matching points in the other vein image are considered unreliable and should be discarded. The whole procedure of MM is as shown in Figure 10.

**Figure 11:**
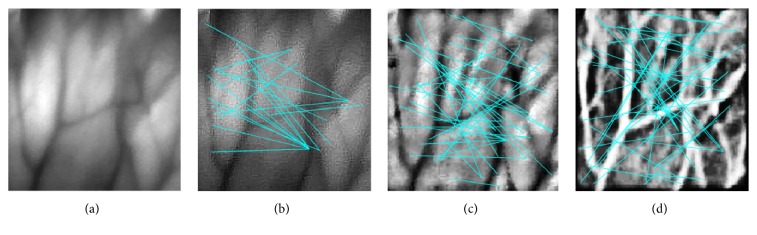
Illumination of MM with random-selected vein images. (a) Original vein images, (b) vein image processed by UM (LNFE), (c) vein image processed by AHE (HEs), and (d) vein image processed by DHE (HEM).

Besides, to fully demonstrate the feasibility of MM in SIFT based vein image matching, random experiments to observe the results in respect to specific CEs of MM with matching points belonging to the same vein images are illustrated in Figure 11, the result of which well shows consistency with the idea of MM.

From the theory description of RootSIFT and MM, an assumption would be formed that the negative influence, brought by CEs, reflecting in bringing in MM for PR and making the OM possible for EER could be solved. The reason lies in that the importation of Hellinger kernel could increase the distinctiveness of feature descriptor, which in potential decreases the MM; and the new matching strategy with MM is capable of discarding the matching points within the same image, which in turn ruin the matching between the vein images belonging to different subjects. The detailed experiment about how the improved SIFT model overcomes the negative influence and obtains state-of-the-art recognition performance is analyzed in the following parts.

### 3.2. Matching and Recognition Experiment with Improved SIFT Model

**Figure 12:**
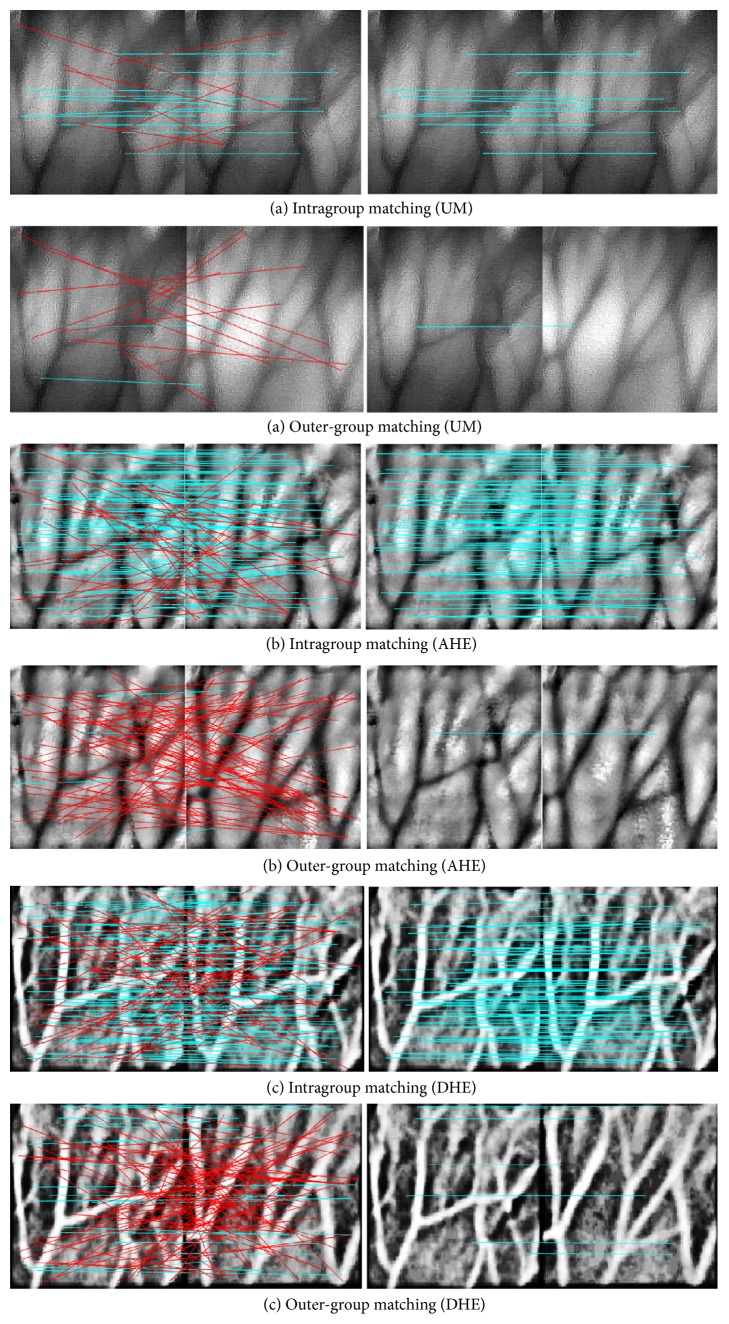
Matching with original SIFT (results on the left) and improved SIFT (results on the right). (a) Vein image processed by UM (LNFE); (b) vein image processed by AHE (HEs); (c) vein image processed by DHE (HEM).

The aim of the recognition experiment is to evaluate how well would the final performance be with improved SIFT model decreasing the negative influence brought by CEs, and the database is the same with the one in Part I. Firstly, Figure 12 shows the mismatching removal effect with the help of Hellinger kernel and MM by random selection of CEs. It is obvious that the mismatching with red line both in IM and OM is all eliminated, and the preserved correct matching with cyan line is well preserved, which indicates a higher PR and EER value.

To fully demonstrate the effectiveness of the improved SIFT model tackling the negative effect of CEs, a comprehensive experiment by adopting all the CEs as the preprocessing method is realized and the corresponding result is as shown in Table 5. It should be noted that the PR value is not listed because the improved SIFT model could cut all the mismatching off, which indicates the highest PR whatever the CE is.

Comparing the EER results with two different databases, it could be concluded that the unified model adopting RootSIFT for keypoints detection and Mirror Matching for keypoints matching could get rid of the negative influence of CE on matching while taking good advantage of CE on improving keypoints detection greatly, and the same trend with two different databases fully demonstrates the generality of the improved model. We also argue that the proposed model could also help improve the performance of other image recognition and matching tasks where contrast enhancement is incorporated for increasing the number of keypoints detection with SIFT algorithm.

**Figure 13:**
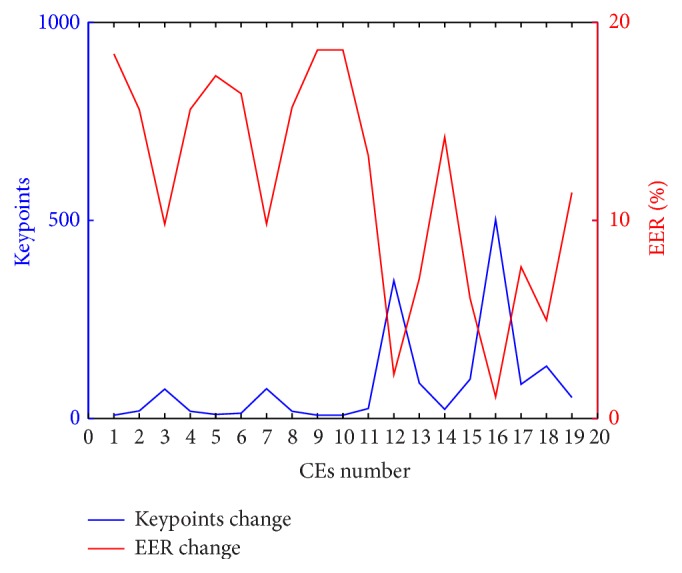
Trend change of keypoints and EER in respect to different CEs. CEs number 1 : 19 represents original, IN, GLS, CS, GC08, LS, UM, HBF, HF, INE, HE, AHE, CLAHE, IHE, DHE, RASF, HHE, and HEHBF, respectively.

What is more, to observe the relationship between change of keypoints and EER, an average increase of keypoints in respect to different CE is obtained and the specific relationship is as shown in Figure 13.

It is obvious that the changing trend of keypoints and EER shows the opposite result, which indicates that the more increased keypoints are brought by CEs, the lower EER it obtains. What is more, we prefer to regard the CE as necessary preprocessing link when aiming to obtain better EER in terms of vein recognition task.

### 3.3. Result Discussion

Bearing the question “is it possible to increase the SIFT based vein recognition performance by incorporating CEs as necessary preprocessing link?” an improved SIFT model with combining Hellinger kernel and MM into an unified framework is proposed to on the one hand remove the mismatching in IM and on the other hand eliminate the mismatching in OM. The experimental result shows that the PR achieves the highest value and the EER could be improved to the state-of-the-art performance with the best one as 1.086%. More surprisingly, it is observed that the keypoints and EER show the opposite changing trend. Consequently, the conclusion that CEs would better the EER of vein recognition system by increasing the number of detected SIFT keypoints unless the mismatching generated by NN matching is eliminated as much as possible is made.

## 4. Conclusions

To show how the CEs will affect the SIFT based vein recognition system, two sets of effective experiments with different hand vein databases are designed: the first one refers to showing that the eighteen CEs yield an increase in the number of SIFT keypoints that can be automatically detected in a vein image, but also a performance decrease in the case of NN matching of generated SIFT keypoint descriptors in respect to PR and EER. However, motivated by the fact that nearly all of the published SIFT based vein recognition systems adopt contrast enhancement as the necessary design, the paper makes attempt to find out how to take advantage of the positive effect of CEs to obtain high recognition performance. Inspired by the experimental result that CEs bring in more mismatching keypoints, an improved SIFT model by designing the kernel of RootSIFT and Mirror Match into a unified framework is realized, and the performance in respect to PR and EER increases a lot, where a state-of-the-art performance with EER as 1.086% is obtained. Besides, further analysis on the relationship between the keypoints increase and EER value change tells us that contrast enhancement is necessary because higher keypoints result in better EER in terms of SIFT based vein recognition system with prerequisite that methods for eliminating the mismatching in IM and OM must be realized.

What is more, in addition to changing matching strategy proposed in the paper, a second model that we consider relevant for highlighting the potential advantages offered by contrast enhancement in terms of increased number of keypoints is vein recognition by using Bag-of-Features (BOF) model [42], which imports the classifier design to obtain the recognition result but not the NN matching. Besides, other matching strategies like Ratio-Match-Ext, Self-Match [43], and so forth could be tried to evaluate the performance. More importantly, developing contrast enhancement techniques that provide an optimal balance between the advantage and disadvantage represents an interesting and for the moment an unexplored research in respect to vein recognition task and other image classification task.

## Figures and Tables

**Table 1:** Influence of selected CEs on the number of detected SIFT keypoints. Values represent the number variations by different CEs on left and right hands with male and female. (The number in the bracket represents the increased times against the number of original images.)

Image set		Linear/nonlinear functional enhancement (LNFE)								
	Original	IN	GLS	CS	GC08	LS	UM	HBF	HF	INE
FL	8	19	74	18	10	13	75	18	8	8
FR	12	19	87	17	9	12	77	17	9	9
ML	13	13	46	13	14	18	93	16	13	13
MR	15	16	53	15	14	17	120	16	15	14

Average	12	17 (1.4)	65 (5.4)	16 (1.3)	12 (1)	15 (1.3)	91 (7.6)	17 (1.4)	11 (0.9)	11 (0.9)

Image set	HE and its variants (HEs)					Hierarchical enhancement models (HEM)				
	Original	HE	AHE	CLAHE	CLHE	IHE	DHE	RASF	HHE	HEHBF

FL	8	25	348	89	23	99	502	86	132	53
FR	12	31	375	25	32	118	438	80	157	53
ML	13	39	299	66	36	134	459	101	137	84
MR	15	40	327	40	38	164	517	82	170	89

Average	12	34 (2.8)	337 (28.1)	55 (4.6)	32 (2.7)	129 (10.8)	479 (39.9)	87 (7.3)	149 (12.4)	70 (5.8)

**Table 2:** Influence of selected CEs on the number of detected SIFT keypoints (PUT Vein Database).

Capturing session		Linear/nonlinear functional enhancement (LNFE)								
	Original	IN	GLS	CS	GC08	LS	UM	HBF	HF	INE
S1	6	19	71	16	11	13	70	19	6	7
S2	11	19	84	15	14	12	72	18	7	8
S3	15	13	43	11	14	18	88	17	11	12

Average	11	17 (1.5)	62 (5.6)	14 (1.3)	12 (1.1)	15 (1.4)	86 (7.8)	18 (1.6)	9 (0.8)	10 (0.9)

Image set	HE and its variants (HEs)					Hierarchical enhancement models (HEM)				
	Original	HE	AHE	CLAHE	CLHE	IHE	DHE	RASF	HHE	HEHBF

S1	6	24	333	103	22	97	449	88	110	52
S2	11	30	350	29	31	116	385	82	135	52
S3	15	38	286	70	35	132	406	113	115	83

Average	11	33 (3.0)	322 (29.3)	59 (5.4)	31 (2.8)	127 (11.5)	426 (38.7)	89 (8.1)	127 (11.5)	69 (6.3)

**Table 3:** Influence of CEs on the Precision-Recall/Equal Error Rate of SIFT descriptor matching. Values represent variations of the PR/EER in respect to the value of the same metric in the absence of CE.

Evaluation item	Linear/nonlinear functional enhancement (LNFE)								
	IN	GLS	CS	GC08	LS	UM	HBF	HF	INE
PR	−2.88	−9.16	−2.46	−1.98	−2.41	−11.76	−3.29	−1.21	−1.07
EER	−0.94	−2.11	−0.70	−0.52	−0.82	−2.25	−1.12	−0.52	−0.34

Evaluation item	HE and its variants (HEs)				Hierarchical enhancement models (HEM)				
	HE	AHE	CLAHE	CLHE	IHE	DHE	RASF	HHE	HEHBF

PR	−8.77	−23.36	−8.69	−8.11	−15.07	−37.29	−10.93	−18.59	−9.19
EER	−1.98	−5.14	−2.05	−1.91	−6.20	−7.97	−2.53	−6.95	−2.39

**Table 4:** Influence of CEs on the Precision-Recall/Equal Error Rate of SIFT descriptor matching (PUT Vein Database).

Evaluation item	Linear/nonlinear functional enhancement (LNFE)								
	IN	GLS	CS	GC08	LS	UM	HBF	HF	INE
PR	−2.93	−8.75	−2.53	−1.36	−3.21	−12.35	−3.12	−1.36	−1.15
EER	−1.05	−3.11	−0.92	−0.47	−0.93	−3.47	−1.21	−0.56	−0.37

Evaluation item	HE and its variants (HEs)				Hierarchical enhancement models (HEM)				
	HE	AHE	CLAHE	CLHE	IHE	DHE	RASF	HHE	HEHBF

PR	−9.21	−27.15	−9.21	−13.15	−36.15	−11.25	−11.03	−19.12	−9.36
EER	−1.76	−6.13	−2.13	−5.12	−8.13	−2.13	−2.12	−6.72	−2.71

**Table 5:** EER value in respect to different CEs on two different hand vein databases.

Database	Linear/nonlinear functional enhancement (LNFE)									
	Original	IN	GLS	CS	GC08	LS	UM	HBF	HF	INE
Lab-made	18.4	15.589	9.8	15.604	17.302	16.4	9.813	15.71	18.605	18.61
PUT	21.3	17.543	8.12	16.954	18.302	15.32	9.672	16.135	19.612	21.36

Database	HE and its variants (HEs)					Hierarchical enhancement models (HEM)				
	Original	HE	AHE	CLAHE	CLHE	IHE	DHE	RASF	HHE	HEHBF

Lab-made	18.4	13.268	2.207	7.056	14.2	6.056	1.086	7.65	4.954	11.4
PUT	21.3	12.736	1.936	7.126	15.1	6.137	0.987	8.352	5.13	12.76
